# Evolution of temperate pathogens: the bacteriophage/bacteria paradigm

**DOI:** 10.1186/1743-422X-4-121

**Published:** 2007-11-09

**Authors:** Arthur L Koch

**Affiliations:** 1 Biology Department, Indiana University, Bloomington, IN 47405-6801, USA

## Abstract

**Background:**

Taking as a pattern, the T4 and lambda viruses interacting with each other and with their Gram-negative host, *Escherichia coli*, a general model is constructed for the evolution of 'gentle' or temperate pathogens. This model is not simply either pure group or kin selection, but probably is common in a variety of host-parasite pairs in various taxonomic groups. The proposed mechanism is that for its own benefit the pathogen evolved ways to protect its host from attack by other pathogens and this has incidentally protected the host. Although appropriate mechanisms would have been developed and excluded related viral species and also other quite different pathogens, the important advance would have been when other individuals of the same species that arrive at the host subsequent to the first infecting one were excluded.

**Results:**

Such a class of mechanisms would not compete one genotype with another, but simply would be of benefit to the first pathogen that had attacked a host organism.

**Conclusion:**

This would tend to protect and extend the life of the host against the detrimental effects of a secondarily infecting pathogen. This leads to the pathogens becoming more temperate via the now favorable co-evolution with its host, which basically protects both host and virus against other pathogens but may cause slowing of the growth of the primary infecting pathogen. Evolution by a 'gentle' strategy would be favored as long as the increased wellbeing of the host also favored the eventual transmission of the early infecting pathogen to other hosts.

## Introduction

Many pathogens are less damaging to their host than they conceivably could be; i.e., they damage the host less than is biologically feasible and replicate at a slower rate than might be possible. The term 'temperate' indicates pathogens that do little or no damage to their host. The term 'lysogeny' refers to the ability of the host cell and virus to enter into the lysogenic state where they grow together and where the virus is functionally hidden in the genome, giving little or no detriment to the bacterial host and is undetectable except with special technique [1]. By definition a lysogenic virus is temperate. On the other hand, viruses that are lethal to their bacterial hosts or cause slower growth are 'virulent'. The two viruses focused on here are at the two extremes. It is not fully self-evident what evolutionary selective force would favor a particular evolutionary stable degree of aggression [2,3]. In the short term such behavior is seemingly counter-productive, but this is not the typical case in nature where a spectrum of strategies are successful. This paradox is an old one, it has been discussed broadly and it has been often actively debated in the specific connection concerning bacteriophages.

Lenski and May [2] analyzed this problem analytically and concluded that the conventional wisdom that parasites and pathogens should evolve to reduced virulence to their hosts is wrong. It was believed that more virulent parasites and pathogens are more likely to drive their hosts, and subsequently themselves, to extinction. Rather Lenski and May conclude that selection will favor whatever level of virulence maximizes the rate of increase of the parasite or the pathogen. This optimization of virulence depends on the functional relationship between a parasite or pathogen's transmissibility and its effect on host mortality. The thesis of their paper is that models in which intermediate levels of virulence are favored lead quite naturally to the further conclusion that parasites and pathogens, only up to a point, should become less virulent over time if the ecological and evolutionary processes are also incorporated into the analysis.

An evolutionary mechanism that could generate pathogens that are neither fully virulent nor temperate is presented here. Such a mechanism was suggested by the knowledge that certain bacterial viruses are known to protect their host from certain pathogens. The prime and oldest known example is the interactions of two quite different pathogens, T4 and λ, of *Escherichia coli*. These coliphages interfere with the growth of each other. Here the relevance of how this phenomenon affects the host's survival is considered and also to suggest a paradigm for the first steps in the evolution of a range of temperate pathogen behavior.

### The 'gentle' pathogen

A temperate virus requires elaborate and delicate controls to modulate its growth and functions in order to respond to the environmental conditions and numbers of its host in the environment. The lysogenic virus must have an ability to function virulently under certain circumstances, and therefore it is not completely gentle. The paradigm of the reproductive strategies of the bacteriophages T4 and lambda has been presented in many ways and in great depth [4-11].

The temperate lambda has genetic mechanisms to protect its host against itself and its near and far relatives. Farther afield, it provides protection against even totally unrelated viruses, such as T4rII. The (common) wild type, T4r^+^, however, has a countermeasure against the lambda offensive. The existence of this elaborate biochemical mechanism is evidence for paths that run counter to the tendency of a hypothetical pathogen in an artificial 'chemostat-like' case under perpetual low multiplicity of infection (moi) conditions to become progressively more virulent toward its host.

### The two-limiting strategies of bacterial viruses and transmissible plasmids

A pathogen at one extreme can operate in an extremely destructive fashion to its host and at the other extreme, it can behave passively towards it host. Both have advantages, but most pathogens are of an intermediate strategy, somewhere in the middle [12].

#### The virulent strategy

When the first pathogen arose that could move from host cell to host cell, it also was a genetic entity that could move a host gene from one organism to another [13]. The transmissible plasmid's or virus's strategy, *a priori*, would be expected to evolve towards almost complete virulence, except for this caveat. In the paradigm of this type of strategy, the virulent pathogen finds a suitable host, exploits it, maximally produces progeny pathogens, the host is destroyed, the descendants escape from the host, and finally they find and parasitize new hosts. Continued selection operates to make all of the steps more efficient and effective as long as the hosts are common and abundant. Coliphages T2, T4, and T6 seem to follow this model almost precisely. Some bacterial pathogens are very efficient in using many parts of the bacterium to make more viruses. Certain bacterial viruses, T2, T4, and T6, not only use the energy generating, enzymatic, and protein synthesizing machinery, but also are so omnivorous that they even consume the host's DNA as a stockpile to achieve even larger virus production [14] Almost every feature of T4 is engineered to capitalize on the bacterial resources. T4 has hundreds of genes and a complex morphology. These features aid the binding, entering, and maximally exploiting a host bacterium. Moreover, they provide many sophisticated regulatory functions to maximize the amount of viral growth.

Other simpler bacterial viruses have fewer genes and a much more streamlined growth strategy, however they can be as virulent. The virulent strategy usually implies using the host machinery and resources to effectively make a larger production of pathogen organisms and incidentally lead to the destruction of the host. From the ecological point of view, as mentioned above, the key for success of this virulent strategy is the availability of a continuing supply of new hosts for further exploitation. Thus the important factor for this pathogenic strategy is that the magnitude of the moi (multiplicity of infection) must be small and a large excess of hosts be available.

#### The temperate strategy

At the other extreme are 'vertically-transmitted' parasites that live in either symbiosis or commensalism with their hosts. In the bacteriophage field, the temperate viruses can achieve the lysogenic state and be propagated by vertical transmission to the next host generation. This implies transmission to both daughter bacteria that are created by cell division. At the absolute (hypothetical) extreme of non-transmissibility of the virus to another host, such a pathogen would have to be strictly non virulent to its current host in order to survive at all. This is the extreme situation. If the pathogen aids the host in some way, then it may be injurious to its host to some limited degree in some other way and still persist. A second possibility, suggested by Ian Molineux (personal communication), is that many vertical pathogens are, in fact, on their way to extinction, but this is happening only very slowly. This loss of virus species is however, balanced by the development of new temperate pathogens of the same class. A large class of plasmids of bacteria is non-transmissible and individuals are propagated from mother to both daughter cells with no transmission to other host organisms. Plasmids are simply a (foreign) group of linked genes that inhabit a cell and propagate therein. When the cytoplasm becomes divided, both daughter cells usually receive at least one copy of the plasmid. While this partition is trivial if the infected cell has many copies of the plasmid, when the average number per cell is only slightly more than one, some special mechanism(s) are needed to sense the cell division event and respond (see the last section of this paper).

It is generally argued that in most circumstances the non-transmissible plasmids are effectively trapped in their host, so they must not destroy or injure it. This implies that they must have clever ways to replicate in synchrony with their host. Moreover, at the same time, they must not upset the growth strategy of their host. The bacterial host, of course, must be able to control its division rate coordinately with its success in converting environmental resources into biomass and this extremely important process must not be interfered with by an internal pathogen if the host and pathogen are both to prosper. If a plasmid can contribute in some way to the fitness of its host, the host may be positively selected. For example, some plasmids confer antibiotic or heavy metal resistance to their host and thus help the host organism evade natural and manmade challenges. At a different level, some genes in plasmids and in prophages help the bacterium in increasing its pathogenicity to a mammalian host. A very good example is the *bor *gene of lambda [15]. It protects the host against serum complement killing. Protecting its bacterial host against the destructive activity of the latter's mammalian host's is a positively selected mechanism.

Although bacteriophage λ has long served as a model system for the study of fundamental biological processes, parts of λ biology remain poorly understood and roughly a third of the λ genome are dispensable for growth and viability under laboratory conditions. These sequences contain numerous open reading frames of unknown function, and their retention in the face of presumably long-standing selective pressures suggests that they provide selective benefits.

These biological situations suggest an evolutionary path of how a 'gentle' state might have arisen in the first place. This is an alternative to the thoughts in the ecological field that are well summarized by Frank [3], which concerns the role that population dynamics plays such as kin or group selection, which might provide mechanisms for the genesis of temperate pathogens. The new model proposed as a possible mechanism appears to be robust and testable.

### The failure of both extremes leading to an intermediate or alternate strategy

The limiting virulent (horizontal) and limiting vertical strategies in their extreme cases are mutually antagonistic. A virulent strategy, when honed by evolution, requires that the pathogen be as avid, as exploitative, and as all consuming to its host as possible in all the ways that will increase the yield of progeny. The non-virulent strategy is the other way around. The parasite should be mild to the point of not doing anything harmful to its host and if additionally it aids the host in some way or ways that would lead to positive selection.

Of course, neither of these extreme strategies will work indefinitely. In a well-mixed continuous culture of a non-mutable single strain of host organism with a non-mutable single strain of an avirulent intracellular pathogen, the pathogen theoretically would not persist because eventually the population of its hosts would be lost by chance or by destruction by another pathogen. The virulent (and transmissible) pathogen would consume all available hosts, and would itself be lost when no more susceptible hosts were available for virus growth. It might survive longer if it was poorly transmissible, but this would be a metastable state because either too much or too little transmissibility would lead to its eventual elimination. Actually the more virulent and highly transmissible pathogens are protected from their own exploitation by environmental heterogeneity [16]. A potential ameliorating circumstance is patchy growth; a particularly good example is wall growth in a chemostat environment or in biofilms generally. Some of the host organisms commonly escape from being parasitized by chance and by this kind of heterogeneity of the environment. That is to say, when the biosphere is composed of separate populations of host organisms, which in some patches may be destroyed by the pathogen, but in other patches, remain temporarily pathogen free and can persist, it is the biological heterogeneity that maintains the pathogen. These can grow, emigrate, and serve as prey for the parasites at various locations at a later time. An additional factor preserving virulent bacterial viruses is that the host can mutate to become resistant to the pathogen. Although the bacterial host can mutate to become resistant to viruses, leading to a population turnover and the near elimination of the pathogen, sensitive revertants usually will later be reselected. Reversion frequently would occur because mounting a resistance mechanism by the host frequently brings with it some selective disadvantage. Such cycling of genotypes causes the regeneration of the sensitive host population and incidentally permits the long-term survival of the virulent pathogen. Under general conditions, Lenski and May [2] showed that intermediate virulence is favored and depends on the functional relationship between the pathogen, its transmissibility, and its effect on host mortality.

With the vertical transmission strategy, the pathogen may persist, especially if it helps its host prosper, but the problem is that the fate of the pathogen in a host organism is conditional on that current host's abilities and on fortune. Thus, the host population in which the parasite is resident might be destroyed by an event entirely independent of the host's or parasite's abilities or coping skills. Thus, strict vertical transmission also is not satisfactory in the long-term and consequently an ability to move to new hosts, even if only needed rarely, is necessary and must be possible in order for the pathogen to persist.

The above points are self-evident and therefore, it is likely that no pathogen successfully employs either extreme strategy. However, the balance point between the two strategies depends a good deal on the population structure and biology of both the pathogen and its host. The important issue is the presence of special additional mechanisms that might be incorporated in the genotype of the host or pathogen or both. For example, benign, but usually non-transmissible plasmids of bacteria need only be transferred occasionally from one host to another for the species to be maintained within the world ecosystem. Such occasional transmissions from the current host to another bacterium can take place under the aegis of a secondary pathogen, which can be a virus or a transmissible plasmid. With this aid, a usually vertically transmitted plasmid can 'piggyback' itself into new organisms without the cost of maintaining a transmission mechanism.

Consequently, the ways that both the mainly virulent and mainly non-virulent strategies do succeed require the possibility of additionally outside intervention or adaptation of a flexible intermediate strategy to achieve long-term persistence, or alternatively, to have in place an appropriate special biological mechanisms. A type of strategy that is used by many pathogens is the alternate use of both of the strategies to various degrees at various times. Many pathogens have master mechanisms that switch them to different limiting strategies under different circumstances.

### The strategy of bacteriophage lambda

Lambda has the ability to alternate between the two ultimate extremes, switching reversibly from one to the other limiting strategy in very sophisticated ways. The control of the switching process is, indeed, elegant [4,6,8,9,17]. Perhaps the most sophisticated and best understood control mechanism in any biological system is the one that allows the lambda to either replicate lytically or to achieve lysogeny and later to escape from the limbo of being a prophage to embark on virulent expansion.

Briefly, a lambda virus attaches, enters the bacterium, select the lysogenic or lytic path. Under suitable conditions, it incorporates its genome into the chromosome of its host. It stays in that prophage state as long as conditions are favorable; in this location its genes are replicated and grow in concert with its host's chromosome. Under stress, the lambda prophage can become virulent, destroying the host bacterium and yielding many synthesized genomes. These progeny virus particles escape from the cell and propagate the species.

In the laboratory, the lytic mode can be switched on by ultraviolet irradiation of the cell, by thymidine starvation, by action of a DNA cross-linker (like mitomycin C), or by a chemotherapeutic agent (like fluorouracil). The molecular mechanisms in the switching process involving *recA *and *l*exA are quite well understood and will not be reviewed here (see [4,11,17]). Temperate viruses prosper while growing as prophages in the genome of a successful host by not harming it and by utilizing the host machinery to only a very small degree with a negligible blockade of it role for the host. However, like 'rats leaving a sinking ship', when conditions for the host are not optimal, the virus not only leaves, but also destroys and uses the resources available within their host to increase the viral yield. The temperate strategy is not uncommon for bacterial viruses in nature and many different viruses lysogenize many different kinds of bacterial cells in this very opportunistic, but reversible way.

### Apparent altruism of lambda

Viruses such as lambda can mutate in several ways to become virulent. These mutants are not able to enter the prophage state, but only grow lytically. This presents the key biological problem alluded to the introduction: Why does the temperate phenotype persist in nature and why does it not become replaced by the virulent form? From simple growth considerations one predicts that the temperate viruses would be rapidly displaced in the population by the 'short-sighted' virulent forms. Lenski and May [2] have analyzed the reason that this prediction is not often fulfilled for the specific case of lambda. The reason is dependent on the genetic mechanisms that the pathogen uses in its interaction with the host. These go well beyond the situation so far described and the details of the mechanism maintaining lysogeny must be appreciated. The key player is the CI protein of lambda (C1 was the second repressor discovered). Its function is to bind to operator specific DNA and prevent all viral genes with only the exceptions of CI, the *rex *genes, and a few others from being expressed. CI must continue to be transcribed, translated, and function to maintain the temperate state and prevent lytic destruction of the cell.

It is the inhibition by CI of the functioning of the majority of the viral genes that is the major protector of the lambda prophage-bearing bacterium in nature. As mentioned, when CI is inactivated or destroyed for any reason virus growth ensues. A prime example is that damage to the bacterium's DNA activates the host's RecA that then acts to cleave LexA, which normally switches on the lytic cycle. This results in death of the cell and the liberation of virus particles. However, there is a second, and probably more important role for CI in the ecology of lambda and its host: The CI protein prevents superinfecting lambda viruses from invading and lysogenizing or growing in the bacterium. Were this not so, virus mutants from the outside would usurp the temperate strain's safe berth and resources, destroy the host, and the original lambda, and generate virulent mutants. Naturally, mutational events generate virulent viruses constantly and the lysogenized bacterium is bombarded with both with them as well as with wild type viruses from the spontaneous induction events (that usually occur about once in a thousand cell cycles). Because of the presence of the CI protein, neither type of exogenous virus is successful in entering and establishing either a lytic infection or the insertion of its genome into the host genome of a previously lysogenized bacterium. (However, vir and super-vir mutants can grow to some degree in lambda lysogens). Thus immunity effectively preserves the prophage genome, and therefore, the first virus gaining entrance into an unlysogenized cell can thwart viruses arriving later. Although the resultant immunity prevents other viral genomes from lytically destroying the host, it allows growth of the host bacterium together with its resident prophage. So this is a mechanism, implemented by the pathogen, which directly aids the host and protects it from outside lambdoid pathogens. It gives lambda's host a selective advantage somewhat equivalent to the cell growth benefit of having, for example, a plasmid with genes for vitamin production, or for resistance to heavy metals, or for resistance to specific antibiotics.

Besides preventing superinfection with exogenous lambda, the resident prophage by making the products of the *rex *genes aids in preventing certain other viruses from invading (see below for details of how these genes block T4r replication). Together, these mechanisms protect the lysogenized bacterium because it impedes the growth of an exogenous virus. All this helps lambda too, but the relevance for the thesis of this paper is that it can favor the development of the 'gentle' pathogen state.

Although lambda can mutate to become purely lytic such that it is not repressed by the transcription and translation of the CI gene of a resident prophage and although such virulent mutations can be made in the laboratory, they only rarely arise in nature. This is because a double mutant would need to be generated: i.e., one that had lost two operators genes, *o*_*l *_and *o*_*r*_. In addition, to the role of two mutations, leading to the probability of the two simultaneous mutations being small, these operator regions are small, and therefore less likely to be inactivated by mutation.

A stable 'gentle' pathogenic strategy results from the virus bequeathing a robust protective action against a range of other potential invaders. These include its own exogenous twin, its one-step mutation, and many-step mutations, as well as other members of the same viral immunity group. The last class is highly significant because many kinds belonging to the lambda immunity group and responsive to CI are present in nature. Finally, the *rex *system acts to protect against still other potential pathogens.

### Lessons from bacteriophage T4

#### The r and r^+ ^phenotypes of T4

Now let us turn to T4 and save further discussion of lambda for later. T4 from nature usually has the r^+ ^phenotype. On the other hand, a mutant of the r phenotype has altered a gene and yields larger plaques on lawns of bacteria spread upon soft agar nutrient plates. The r designates the 'rapid' lysis phenotype; it has the rIIA or rIIB mutation or both of these, or still others genetic changes. The r^+ ^wild type forms very small plaque because the time from infection to lysis of a cell is prolonged. This effect is called the 'lysis-inhibited phenotype' (LIN). I (developed a theory of plaque enlargement rate [18]; this mathematical analysis shows that two factors are important in determining the enlargement rate of the radius of the plaque. These are the mean time from infection to lysis and the diffusion constant for the free virus in the environment. Abedon *et al*. [19] have studied these processes experimentally. They found that phage RB69 (similar to T4) when grown at high bacterial densities, produced mutants, such as *sta5*, which have adapted to have very short latent periods. Although it might be thought that the rapid-lysis phenotype would take over every population of T4 viruses in the world just the opposite is found. This initially unexpected result can be understood on several bases. First, because the cell and virus concentration and/or the multiplicity of infection (moi) typically are low or patchy in nature and there is little advantage to either form. Although the r^+ ^variety has a very long latent incubation period (LIN), the LIN phenotype only happens when additional copies of the viruses infect a cell after the initial infection. In this situation when lysis does eventually occur, a larger burst size is produced. While this may be important in a fluid environment, however, in an unstirred medium, or in a dense culture, or on the surface of an agar plate, or a turbid viscous suspension, the critical factor is a short time for lysis. In contrast, when the bacteria are only singly infected with r^+ ^virus they lyse at the same time as do cells infected with the r form. Moreover, both virus types yield the same size burst (yield) of virus, in single infection and therefore, neither is at an important disadvantage with the other. The second factor, the lysis-inhibition mechanism, functioning in r^+ ^infected cells only acts when a superinfecting virus triggers the lysis-inhibiting response. Quite importantly, when this mechanism is turned on, the secondarily infecting virus particles are destroyed in the periplasmic space.

Under the rapid lysis conditions, two proteins are important: lysozyme e (or gt*e*) and holin (t or gtt). The latter creates a pore in the cytoplasmic membrane (after about 25 to 30 min) so that the lysozyme can enter the periplasmic space from the cytoplasm, breakdown the murein wall, and thereby, permit the liberation of the virus.

The r^+ ^mechanism for control of 'lysis inhibition' (LIN) has not been completely elucidated. The timing of the holin action is critical. It can be delayed in the presence of r^+ ^for at least six hours while more viruses are completed. It is known that this delay depends on a membrane-bound and on a cytoplasmic protein. However, what in addition is involved and how time is 'kept' is not clear [1,20,21]. In sum, wild type T4r^+ ^virus has a mechanism so that after superinfection a delay in lysis occurs and causes a larger yield of phage with the genome of the primary infecting particle, and does not use the secondarily infecting one. This occurs because it destroys the genomes of secondarily arriving viruses, even of those with exactly its own genotype. This gives the r^+ ^virus an advantage over the r form in spite of its seeming disadvantage during growth on a 'lawn' or 'biofilm' of bacteria during a plaque assay (as on a nutrient agar Petri plate). From the viewpoint of the proposed model, it is a mechanism that favors the genotype of the first infecting virus, just as in the lambda case, and it gives the bacterial cell a longer lifespan before its destruction.

### T4 versus lambda

The above is not a full description of the way in which the rII-system functions. However, how does it prevent the rapid selection of the r genotype in favor of the larger yield of progeny of the lysis-inhibited r^+ ^infection? To understand this we have to consider a second, but related, process; i.e., the interaction between the prophage of lambda and an invading T4. Exploiting this interaction was fundamental to the important conceptual advance of Benzer [4] leading to the definition of the 'cistron', as the smallest unit of DNA that coded for a gene function. This in turn led to the modern definition of the gene. Benzer's experiments depended on the fact that T4 rllA and T4 rIIB mutants would not grow on an *E. coli *K-12 strain that was lysogenized with lambda, but would grow on *E. coli *strain B or a prophage-free strain of K-12. This experimental system was key to also permitting Crick *et al*. [7] to establish the protein code as 'commaless' and led to the concept of codons formed of groups of three nucleotides that together specified the specific amino acids.

#### Viral 'apoptosis'

The system of K-12/lambda/T4r versus K-12/lambda/T4rll was the first well-studied case where one virus aided the host against another kind of virus. Lambda has genes, *rexA *and *rexB*, which map adjacent to CI. They are like CI, but unlike almost all other viral genes in the prophage state, in that they are expressed. The RexB protein becomes localized in the cytoplasmic (inner) membrane. It is believed, but not fully proved, that this protein is for a channel (or a gene that controls one). When the pore is opened, it can destroy the cell by allowing exogenous ions to enter. When open, Na^+ ^ions flow into the cell and the bacterium is killed. The point is that because these proteins are continuously synthesized, these two rex proteins constitute a mechanism that is always ready to keep T4r from multiplying. Because the resident lambda and the host cell are destroyed in the process this is a clear case of biological 'apoptosis' or 'apparent altruism' [22]. Ecologically, it would be argued that this suicidal event allows the lambdas that had lysogenized bacterial cells, which had not happened to become infected with T4r to have a smaller chance of becoming infected with T4r. This allows those host cells to keep on growing and propagating lambda prophages. This behavior is altruistic and an elegant case of kin selection and has survival value for both the virus and the host bacterial species. It should be noted that many of lambda close relatives do not have this protection and do not exclude rII mutants.

Quite common in biological systems is the phenomenon of kin selection for self-destructive behaviors. For example, it is how plant cells often respond to infections: the cells surrounding an infected cell die in response to a cell-programmed process, and this prevents the infection from spreading and becoming systemic. There is a process (in bacteria) that is almost the reverse, but is destructive of the host cell for the indirect benefit of the plasmid pathogen. In this case certain small copy-number plasmids have a mechanism of making a protein that can kill the host bacterium. Ordinarily the mechanism is inactive because the plasmid blocks its action. If by chance the plasmid-bearing cell divides to yield one daughter that is free from the plasmid, the killing mechanisms function because the inhibitory protein is no longer present. This maintains the plasmid-bearing line. (See *Related Matters*). Other parallel behaviors could also be cited.

In the laboratory, the suicide mediated by *rexB *of lambda-containing *E. coli *when secondarily infected by T4rII can be prevented or ameliorated by treatment with high levels of Na^+ ^or with significant, but lower levels of Mg^++^. Sucrose and polyamines will also protect the cell. The RexA protein is a cytoplasmic protein. It somehow serves to sense the intruding T4rII virus and activate the RexB destruction function. These lambda proviruses exclude not only T4rII, but also many other viruses. (This, apparently, may not include superinfecting lambda viruses; these are prevented from growing by the CI repressor protein coded by the resident prophage). It may be that other viruses that may have little or no relationship to each other, to T4, or to lambda, but may trigger the RexAB switch leading to total destruction of the cell and, of course, the viral genomes contained therein. For the world ecosystem as a whole, this favors the host and the resident lambda prophage.

This is not the end of the interactions between these two viruses. To successfully infect a cell bearing a lambda prophage, a virus such as wild-type T4r^+ ^has a counter-measure against lambda's exclusion mechanism. This counter-counter-measure is the r^+ ^system. T4 with an intact r^+ ^gene can grow in K12-bearing lambda prophage, and it is the loss of this special mechanism that accompanies the change of T4r^+ ^to T4rII by reactivating A or B, or both. The mode of action of these genes is still not clear. Thus T4r^+ ^has a way to fend against the lambda's RexA/RexB system so it can enter a lysogenized cell, replicate, and destroy the host genome together with the resident lambda's prophage. This is the third, and possibly most important, reason why T4r^+ ^is the form of T4 generally found in nature. The RexAB system of lambda has other effects as well. The RexAB system of lambda has other effects as well. Thus, Bockrath's group [23,24] found that this system affected the photolyase system. This circumstance has further implications that are unexplored.

## Conclusion

From these complex examples of interactions between a host and different kinds of viral pathogens an important group of potentially general principles can be drawn. In other cases that could have been considered the specific biochemical mechanisms might be quite different while the biological outcome can be the same; i.e., the maintenance of the diversity of hosts and predators. For the highly evolved system of species of hosts and pathogens considered here, the following conclusion can be drawn:

(i) Viruses that are unusually 'gentle' have genetic mechanisms to protect their host, at least temporarily, against themselves (lysogeny or its equivalent).

(ii) Some pathogens provide protection against the action of later arriving pathogens of the identical kind (or of near relatives).

(iii) Some pathogens may provide protection against even totally unrelated viruses by special mechanisms. These special ways may involve altruistic (kin) selection such as killing themselves together with their hosts to prevent the growth of another kind of virus. This protects other nearby uninfected hosts from infection.

(iv) They may provide protection against pathogens that evolved earlier (in earlier eons), but are not common now. However, the genetic memory is still there and still would be able to afford protection.

The protective mechanism of lambda against T4r may have led to T4r^+ ^superseding T4r. One can be sure that this particular system of the lambda and T_even _interaction is only one of many elaborate and sophisticated sets of biochemical mechanisms that accomplish the measures and the countermeasures permitting survival of all under suitable conditions. This suggests that many pathogenic organisms have evolved mechanisms for just this purpose in the past. While many are not altruistic, some host destructive behaviors are altruistic in that they are bad for the individual parasite but good for the species of the host. This is the key point here; i.e., they may be favorable for their host population. These cases are just as altruistic as in the case in which a worker bee aids the hive and the genes in the queen, her own sister, by stinging an intruder in spite of the fact that her act will mean her own death. In each case, the genome prospers within the group, although the individual does not. It is clear that some pathogenic species are able to protect their host against other pathogens not for their own direct benefit, but an eventual benefit to their own genome.

### Overcoming the tendency to become more virulent

On the short time scale, a pathogenic organism could be expected to evolve to become more virulent even though that strategy would be counter-productive at the end. It is certainly at first surprising that many organisms with temperate strategies have persisted. I feel that such restrained and altruistic behavior did not arise in many cases by mechanisms of population dynamics, such as by group or kin selection. The literature is not convincing that any of the mechanisms are sufficiently powerful or robust enough to have generated so many 'gentle' pathogens as in the world's biota. I feel this way in spite of the large body of papers by early and current authors, as summarized in the review by Frank [3] and the above. Instead, it is suggest that via the evolution of mechanisms that prevented secondary infections by other pathogens is the basis for incidentally generating 'gentle' pathogens entirely by direct positive selection.

### A plausible sequence for the initial generation of a 'gentle' pathogen

It is reasonable to assume that plasmid-like non-transmissible pathogens preceded the development of a genetic vehicle to move genes from cell to cell [13]. This type of pathogen could only contain a few of the genes from the host and must have been quite different from modern plasmids and viruses. It can be imagined that subsequent to the development of cell-to-cell transmission mechanisms that fully virulent viruses developed and were the primitive type of extracellular pathogens that could be transmitted occasionally from host to host. At this stage of evolution, pathogens had developed ways to infect a cell, grow within it, and eventually lyse or kill its host to liberate virus particles that could spread through the environment and enter other cells to repeat the cycle.

A hypothetical sequence leading from virulence to non-virulence or equivalently from rapid and complete destruction of the infected host to its partial preservation in the infected state can be constructed from considerations of the properties of only the pathogens of *E. coli *discussed above. The proposed evolutionary sequence leads from purely virulent viruses like Qβ or T4r to various intermediates like the lambda, but missing the *rex *system. Next in the sequence are fully temperate viruses like wild type lambda.

As such viruses evolved to become more efficient, and at more times and places, their growth would have become limiting by the availability of suitable hosts. However, it would become a net advantage instead of a disadvantage if a virus prevented other viruses from entering the host and taking over. It could then take longer in the replication phase and producing a higher burst size. The optimum strategy would have as a result been changed and now it is to prolong the life of the resident host.

Possible types of mechanisms to protect a virus include the two that have already been discussed, the equivalent of the CI protein, and the wild type rII^+ ^proteins. The former prevents secondary viruses of the same kind (or incompatibility group) from invading and replacing it. The latter proteins prevent the concomitant growth of a variety of non-related pathogens. Possibly, the other known r systems, rI, rIII, and rIV genes in their wild type forms are equivalent, but may have different ways to protect the resident virus against other ranges of viruses. It must be reiterated that both the CI and rII proteins are inhibitory to the genome of the cell that created them, but they aid the host population by either inhibiting the growth of the resident pathogen or by killing their host to favor the survivorship of its close relatives in nearby hosts. In the highly evolved state of today's lambda, which could have resulted from an early infecting virus that took refuge in the host's DNA as a prophage, it is an advantage. Even before the mechanism for incorporation of the viral genome into the host genome developed, however, the CI immunity protein might slow the growth of the first arriving virus and also prevent the growth of the superinfecting virus. In such a haven, such earlier pathogens have some advantage if they grow slowly, but more abundantly.

The ideas presented here, although couched in the lore of prokaryote pathogens, may have important implications for diseases caused by pathogens in organisms in general. Not only are the implications for HIV-disease obvious, but also the application of these concepts to a range of diseases and some mechanisms of innate resistance is also evident. Even if we do not know the analogues of the genes discussed above, they may exist or have existed and have led to the stage where the contemporary temperate pathogen is a most successful form.

It may be appropriate to end by drawing an analogy to the extended biological role of the MHC (or HLA) part of the vertebrate immune system. Some of the disadvantages of several of the large number of extant alleles of the MHC system are now known. For example, the B27 allele which is associated with ankylosing spondylitus; DR2 with the Goodpasture syndrome and multiple sclerosis; and DR3 and DR4 with Type I diabetes. These are the downside of these genes, but many workers believe that these various alleles give an advantage to their possessor under some (but generally unknown) circumstances. These hypothetical advantages are postulated to explain why these immunological alleles exist. These postulated advantages of these alleles must be so strong that they overweigh the known detrimental aspects. However, in only few cases do we know what the advantage actually is (or was). In contrast, we know the disadvantage of having genes contributing to human disease, like schizophrenia. Because of the inheritance modes, because of the wide spread and common occurrence in many ethnic groups of this disease, one can believe that these genes must provide, or have provided, some important advantage, such as protection against certain diseases, but again we are not yet sure what the diseases really are. Malaria and Sickle cell anemia are an additional case that is relevant here. Similarly, we and other organisms may be protected from many diseases by having many other diseases that are so 'gentle' that we do not know of their presence, but which are actually protective.

### Related matters

We have been considering a way by which a virus or a plasmid can destroy itself but help the population and its species at large and incidentally help the host to survive when attacked by another virus or transmissible plasmid. But the cases specifically discussed here do not cover all the relevant ways that the pathogens of prokaryotes end up helping their host by their own behavior and delay their host's destruction, and thereby increase the survival of the species of host that they depend on and indirectly their own species.

The mechanism (so far un-discussed) covering one important case can be called, any of the following – an addiction module, programmed cell death, stress response, toxin-antitoxin, control, or just TA. These are different terms for the idea that the cell under the aegis of the pathogen makes a toxin, but then protected itself from it by an antidote usually designated as an 'antitoxin'. The purpose of this is to protect the cell – except for the case when the pathogen has been eliminated. Then, the cell dies because the antitoxin become eliminated more rapidly that the toxin. It is argued that the whole purpose of the mechanism from the point of view of the pathogen is to eliminate pathogen-free cells. This allows the population of pathogen-containing cells to survive a competition of pathogen-free cells that probably would grow faster; this indirectly leads to the benefit of the pathogen and incidentally to the benefit of the host. In this way the AT mechanisms are like the cases discussed here in which the host prospers at the immediate expense of some pathogen.
